# Therapeutic role of uterine-derived stem cells in acute kidney injury

**DOI:** 10.1186/s13287-022-02789-0

**Published:** 2022-03-12

**Authors:** Ramanaiah Mamillapalli, SiHyun Cho, Levent Mutlu, Hugh S. Taylor

**Affiliations:** 1 Department of Obstetrics, Gynecology and Reproductive Sciences, Yale School of Medicine, 310 Cedar Street, New Haven, CT 06510 USA; 2 Present Address: Department of Obstetrics and Gynecology, Gangnam Severance Hospital, College of Medicine, Yonsei University, Seoul, South Korea

**Keywords:** Acute kidney injury, Uterine-derived cells, Renal function, Survival, Hysterectomy

## Abstract

**Background:**

Acute kidney injury (AKI) causes abrupt deterioration in kidney function that disrupts metabolic, electrolyte and fluid homeostasis. Although the prevalence of AKI is steadily increasing, no definitive treatment options are available, leading to severe morbidity and mortality. We evaluated the role of uterine-derived multipotent stem cells in kidney regeneration after ischemic AKI.

**Methods:**

Female C57BL/6J mice were hysterectomized and subsequently subject to AKI by either unilateral or bilateral renal ischemia–reperfusion injury. Uterine-derived cells (UDCs), containing a population of uterine stem cells, were isolated from the uteri of female transgenic DsRed mice and injected intravenously to AKI mice. Engraftment of DsRed cells was analyzed by flow cytometry while serum creatinine levels were determined colorimetrically. Expression of UDC markers and cytokine markers were analyzed by immunohistochemical and qRT-PCR methods, respectively. The Kaplan–Meier method was used to analyze survival time while unpaired *t* test with Welch’s correction used for data analysis between two groups.

**Results:**

Mice with an intact uterus, and hence an endogenous source of UDCs, had a higher survival rate after bilateral ischemic AKI compared to hysterectomized mice. Mice treated with infusion of exogenous UDCs after hysterectomy/AKI had lower serum creatinine levels and higher survival rates compared to controls that did not receive UDCs. Engraftment of labeled UDCs was significantly higher in kidneys of bilateral ischemic AKI mice compared to those that underwent a sham surgery. When unilateral ischemic AKI was induced, higher numbers of UDCs were found in the injured than non-injured kidney. Immunofluorescence staining demonstrated double-positive DsRed/Lotus tetragonolobus agglutinin (LTA) positive cells and DsRed/CD31 positive cells indicating contribution of UDCs in renal tubular and vascular regeneration. Expression of *Cxcl12*, *Bmp2*, *Bmp4*, and *Ctnf* in renal tissue was significantly higher in the UDCs injection group than the control group.

**Conclusions:**

UDCs engrafted injured kidneys, contributed to proximal tubule and vascular regeneration, improved kidney function and increased survival in AKI mice. UDC administration is a promising new therapy for AKI. Endogenous uterine stem cells likely also preserve kidney function, suggesting a novel interaction between the uterus and kidney. We suggest that hysterectomy may have a detrimental effect on response to renal injury.

## Background

Acute kidney injury (AKI) is a cause of chronic kidney disease, impacting more than 13 million patients annually with a death toll of 1.7 million globally [1]. It is also a risk factor for development of cardiovascular diseases [2]. AKI affects one in five hospitalized patients and has a mortality rate exceeding 25% per year [3]. The community-based incidence of AKI is also steadily increasing [4]. AKI leads to a deterioration in kidney function that disrupts metabolic, electrolyte and fluid homeostasis over a period of hours to days. The annual health care expenditures attributable to hospital-acquired AKI are estimated to exceed $10 billion [5]. Although AKI is associated with high morbidity and mortality, no specific treatment strategies have been introduced other than management of the underlying cause and providing supportive care [6]. As conventional treatments have failed to improve treatment of AKI, focus has shifted to regenerative medicine as a new therapeutic option. Animal models of renal failure have demonstrated that human fetal nephron progenitor cells participate in repair of renal tubules [7]. The therapeutic effects of human induced pluripotent stem cells (hiPSCs) on AKI have been reported in experimental mouse models [8, 9].

Recently, the endometrium has emerged as an accessible source of adult stem cells with remarkable differentiation capacity. The human endometrium is a highly dynamic tissue that is completely regenerated in approximately 400 menstrual cycles during a woman’s lifetime. It is known to contain a robust population of stem/progenitor cells [10–12]; characterization of endometrial stromal cells has identified a population of mesenchymal stem cells that can differentiate into adipogenic, chondrogenic and osteogenic fates in vitro [12, 13]. Since then, numerous therapeutic applications using endometrial stem cells to treat non-endometrial diseases have been introduced. Differentiation and transplantation of human endometrial stem cells have been shown to increase concentrations of dopamine and its metabolites in mouse and non-human primate models of Parkinson’s disease [14, 15]. Insulin-producing cells have been generated from endometrial stem cells, and their therapeutic potential has been demonstrated using a diabetic mouse model. Transplantation of human endometrial stem cell-derived beta-like cells improved hyperglycemia in the recipient mouse as well as prevented diabetes-associated complications [16]. The uterus has also been shown to be a potential stem cell source for the treatment of heart disease. Human menstrual blood-derived mesenchymal stem cells have been successfully differentiated into spontaneously beating cardiomyocyte-like cells and injection of these cells into infarcted zones in the heart of a murine model induced functional improvement with smaller infarct areas [17].

We initially observed a higher survival after AKI in animals with an intact uterus compared to those that underwent hysterectomy and hypothesized that uterine stem cells may contribute to renal tubule regeneration. Although applications of endometrial stem cells have been suggested as a therapeutic option in multiple diseases, there are few studies evaluating their potential in the treatment of AKI. Based on the fact that the uterus and kidney share the same embryological origin, we hypothesized that uterine-derived cells (UDCs) may be participating in kidney repair. In this study, we utilized an AKI mouse model of renal ischemia–reperfusion injury and injected UDCs into the circulation. We first evaluated whether UDCs can engraft the injured kidney. Then, we evaluated the potential use of UDCs for AKI treatment using kidney function testing and survival analysis. We find an unsuspected role for the uterus and uterine-derived stem cells in repair of the kidney after ischemic injury.

## Materials and methods

### Animals

Female transgenic DsRed (Discosoma sp. Red Fluorescent Protein, Strain #009655, C57BL/6J-Tg (Dcx-DsRed) mice (8-week-old) from Jackson Laboratories (Bar Harbor, ME, USA) and female C57BL/6J mice (8-week-old) from Charles River Laboratories (Wilmington, MA, USA) were purchased and maintained under controlled conditions (a 12-h light, 12-h dark cycle, at 22 °C) with free access to water and chow. Animals were treated in accordance with a protocol approved by the Yale University Institutional Animal Care and Use Committee (IACUC), and following the U.S. Principles for Utilization and Care of Vertebrate Animals Used in Testing, Research, and Training.

### Hysterectomy

Mice were anesthetized with inhaled isoflurane and received 1.0 mg/kg meloxicam, 1.2 mg/kg buprenorphine. All mice received a ventral midline incision through the skin and peritoneum. The sham surgery group received skin and peritoneal incisions only. In the hysterectomy group, each uterine horn was ligated and cut below the ovary and oviduct. The uterus was then separated from the adjacent fat, and the uterocervical junction was ligated and cut above the cervix. All incisions were sutured with dissolvable vicryl suture, and mice were allowed to recover under a heat lamp and were single-housed for 7 days following the surgery.

### Ischemia–reperfusion model

Mice were given pentobarbital (60 mg/kg, Sigma, St Louis, USA) and then heparin (100 units) by intraperitoneal injection and kept on a heated surgical pad at 37 ± 1 °C. A cannula was inserted into the right femoral vein to infuse normal saline at 3 ml/h and also administer bolus doses of pentobarbital sodium as required. Another cannula was placed into the right femoral artery and connected to a pressure transducer (MLT844; ADInstruments, Bella Vista, NSW, Australia) for continuous recording of arterial pressure by means of a PowerLab/4SP data acquisition system (AD Instruments). A midline electrosurgical laparotomy was performed to carefully separate the renal artery and vein from each other on both sides under a surgical microscope (SZ2-ET; Olympus Corp., Tokyo, Japan). Following a 30 min equilibration period, either one or both renal arteries and veins and pedicles were occluded using non-traumatic clamps for 30 min, during which arterial pressure and heart rate were recorded by the PowerLab. At 5 min before finishing the renal ischemic period, the urinary bladder was punctured to completely discharge urine and then cannulated. Mice body temperature was controlled at 36.8–37.2 °C during surgery with a temperature-controlled operating table (Vestavia Scientific). After release of clamps and confirmation of reperfusion by return of normal kidney color, an immediate 30-min clearance period was undertaken. During the clearance period, urine was collected. A separate group of mice were maintained for immunohistology studies. At the end of ischemic period and clearance period, mice in this group had an arterial blood sample (1 ml) taken and quickly centrifuged, and then, both kidneys were removed and the mice were euthanized by infusion of a concentrated KCl solution. The sham group had all surgical procedures and blood sampling but without induction of renal ischemia. Each group consisted of 8 animals. Unilateral kidney injury in female mice to determine the percent of DsRed cells engrafted into the injured vs uninjured kidney.

### Preparation of UDCs for in vivo treatment

8–12-week-old female mice (C57BL/6) expressing red fluorescent protein (DsRed) were used as donors. These mice were euthanized under anesthesia as described above; the uteri were harvested, stripped of all adjacent adipose tissue, and digested in a solution of Hank’s balanced salt solution (HBSS) containing DNAse I (0.085 mg/ml) and collagenase B (0.85 mg/ml). Cells were counted using trypan blue by hemocytometer and the cell pellet was washed twice and suspended in PBS. One million (1 × 106) UDCs were injected through intravenous (i.v) injection immediately after kidney injury.   

### Kidney imaging by fluorescence

Kidneys were removed from both the control and UDC groups, and imaged with Carestream In Vivo MS Fx Pro (Carestream Health, USA) to capture X-ray and corresponding fluorescent images of the uterine cells that engrafted the kidney.

### Flow cytometry

Kidneys were removed from both the control and UDC treated groups of mice and assessed to determine the presence of DsRed + cells using fluorescence-activated cell sorting (FACS). Kidney tissue was finely minced with surgical scalpels and subsequently digested with a solution of Hank’s Balanced Salt Solution (Life Technologies, Inc, Invitrogen, Carlsbad, CA) containing HEPES (25 mM), collagenase B (1 mg/ml, Roche Diagnostics, Indianapolis, IN), and deoxyribonuclease (0.1 mg/ml, Sigma-Aldrich, St. Louis, MO) for 60–90 min at 37 °C. All samples were filtered using 70-μm cell strainers and centrifuged at 3000 rpm at 4 °C for 5 min. Cell pellets were suspended in FACS buffer and then sorted via FACS on a Beckman Coulter MoFlo machine (Beckman Coulter, San Jose, CA). Acquired data were analyzed using the FlowJo V10 software (Tree Star, Ashland, OR) for three independent experiments.

### Immunofluorescence studies

Kidney tissue was fixed in 4% paraformaldehyde and embedded in paraffin. Five-micrometer tissue sections (5 µm) were mounted on slides, steamed in sodium citrate at pH 6 for 10 min for antigen retrieval, and blocked using 10% donkey serum (Vector Laboratories, Burlingame, CA, USA). Sections on slides were incubated at 4 °C overnight with primary antibodies, rabbit anti-CD31 polyclonal antibody (catalog #ab28364 Abcam, Cambridge, MA, USA), goat anti-DsRed polyclonal antibody (catalog # sc-33354, Santa Cruz Biotechnology, Dallas, TX), and rat anti-CD45 antibody (catalog # ab23910, Abcam, Cambridge, MA) and anti-LTA monoclonal antibody (catalog # 10R-1174, Fitzgerald Industries International, North Acton, MA, USA). Secondary antibodies used were Alexa Fluor 568-conjugated donkey anti-goat (1:200, catalog #A11057, Invitrogen, CA, USA) and Alexa Fluor 488-conjugated donkey anti-rabbit (1:200, catalog #A21206, Invitrogen). Sections were mounted under coverslips using Vectashield fluorescent mounting media with 46-diamidino-2-phenylindole (DAPI) (catalog #H-1200; Vector Laboratories, Burlingame, CA). Visualization of the slides was performed using a laser scanning confocal microscope (LSM 710; Zeiss) and the ZEN software (Carl Zeiss).

### Quantitative real-time polymerase chain reaction (qRT-PCR)

RNA was extracted from kidney tissue by TRIzol reagent (Life Technologies), followed by purification using the Qiagen cleaning kit (Qiagen, Valencia, CA, USA) to prepare cDNA with 50 ng RNA in a 20 μl reaction mixture by iScript cDNA Synthesis Kit (Bio-Rad Laboratories, Hercules, CA). Quantitative real-time PCR was performed to quantify *DsRed*, *Cxcl12*, *Bmp2*, *Bmp4*, and *Ctnf* mRNA expression. The specificity of the amplified transcript (39 cycles) and absence of primer-dimers was confirmed by a melting curve analysis. Gene expression was normalized to the expression of β-actin for each sample. Relative mRNA expression for each gene was calculated using the comparative cycle threshold (Ct) method, also known as the 2^−ΔΔCT^ method. All experiments were carried out three times and performed in duplicate. Nuclease-free water was used as a negative control replacing the cDNA template.

### Serum creatinine estimation for renal function

Blood samples were collected on day 0, 1, 2, 4, 7, 14 and 21 from both the control as well as UDC groups, and serum was collected by centrifugation at 5000 rpm for 10 min at 4 °C and frozen until used. Serum creatinine levels were determined colorimetrically using creatinine assay kit purchased from Abcam (catalog #: ab64340, Cambridge, MA, USA). Mice serum creatinine levels were determined by extrapolating the OD values from the standard curve.

### Statistical analysis

Statistical analysis was performed using SPSS 16.0 (SPSS Inc, Chicago, IL) or GraphPad Prism 6 software (GraphPad Software). Mean total cell counts and creatinine levels between the groups (control vs. UDC; male vs. female; injured vs. non-injured) were compared using an unpaired t-test with Welch’s correction for unequal variances. The expression levels of mRNAs between the two groups were also compared using an unpaired t-test with Welch’s correction. The Kaplan–Meier method was used to analyze survival time between the two groups. *p* < 0.05 was considered statistically significant, and the data were expressed as mean ± SEM.

## Results

### Prior hysterectomy decreased survival after renal injury

**Fig. 1:**
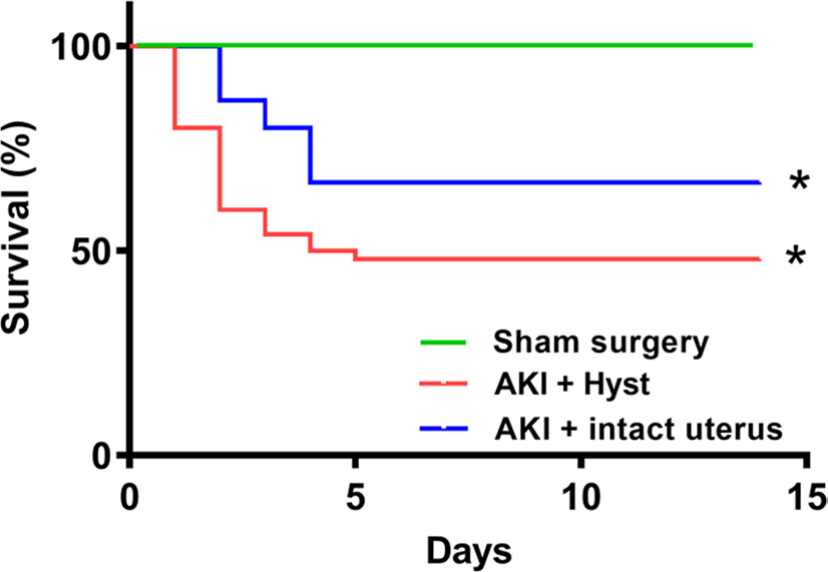
Survival of mice with or without hysterectomy**.** All mice survived in the sham surgery. Survival in all mice after ischemia–reperfusion acute kidney injury (AKI) was lower than sham surgery controls. Mice without hysterectomy (intact uterus, blue) had a higher rate of survival compared to those with hysterectomy (Hyst, red). *N* = 8 mice per group. *denotes statistical significance (*p* < 0.05) comparing AKI + Hyst or AKI + intact uterus vs sham surgery as well as comparing AKI + Hyst vs. AKI + intact

Survival rate after AKI via bilateral renal ischemia–reperfusion injury was compared between hysterectomized mice and those with intact uteri. Mice with intact uteri had a significantly higher survival rate after 2 weeks compared to that in the hysterectomy group (Fig. 1). Sham surgery or hysterectomy alone without induction of AKI had no effect on mortality. This indicated a potential beneficial role of the uterus in recovery from AKI and in overall survival.

### UDC engraftment of the injured kidney

**Fig. 2:**
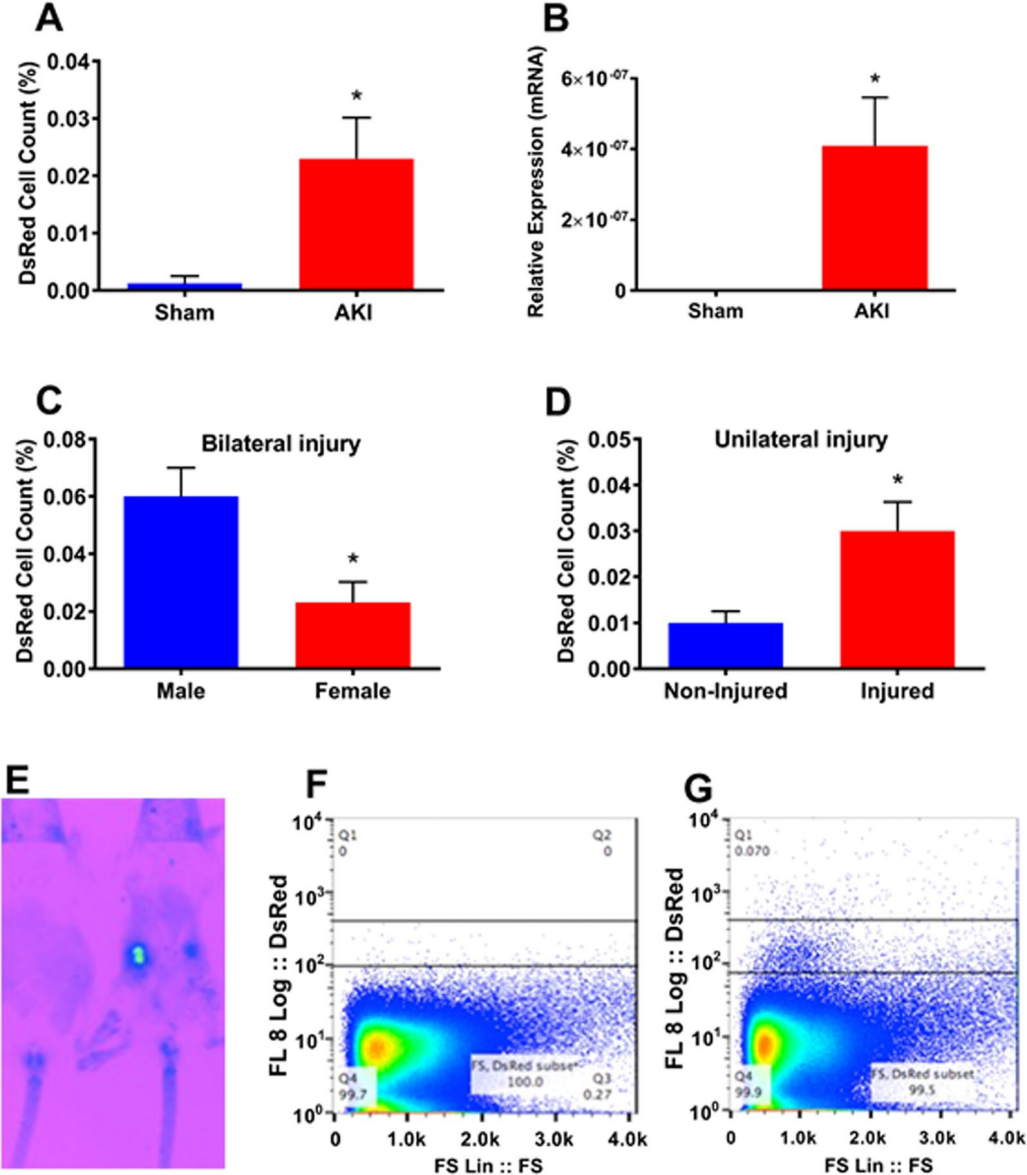
Labeled UDCs engraft the injured kidney as demonstrated by cell count, renal DsRed mRNA expression levels, in vivo imaging system and fluorescence-activated cell sorting (FACS). **a** Ds-Red positive uterine-derived cells (UDCs) were injected immediately following either sham surgery or bilateral renal ischemia–reperfusion injury. DsRed positive cells were preferentially incorporated into the injured kidneys as determined by FACS. **b** Similarly, elevated DsRed mRNA expression levels were higher in the kidneys of the renal ischemia–reperfusion injury group compared to the sham surgery group. **c** DsRed positive cell counts are shown in either male or female kidneys after ischemia–reperfusion injury. DsRed cells were preferentially incorporated in males. **d** After unilateral ischemia–reperfusion injured, more DsRed positive cells accumulated in the injured kidney compared to the non-injured kidney. **e** In vivo imaging of sham group and unilateral renal ischemia–reperfusion injury. DsRed positive uterine-derived cells (UDCs, shown here in green) were preferentially incorporated into the injured kidney. **f** and **g** demonstrate fluorescence-activated cell sorting of uninjured and ischemia–reperfusion injured kidneys. **f** DsRed labeled uterine-derived cells (UDCs) were not incorporated into the uninjured kidney in significant numbers. **g** Large numbers of DsRed cells were incorporated into the injured kidney. (*N* = 8 per group) *denotes statistical significance (*p* < 0.05) sham vs UDC; male vs female and non-injured vs injured

Our previous studies have characterized a population of resident multipotent stem cells in the uterus; specifically, these cells reside in the uterine endometrium. We have demonstrated the ability of labeled human endometrial stem cells to migrate to the site of an injury, engraft, differentiate in vivo*,* and significantly improve function in other organ systems [14, 15]. Here, we next evaluated whether UDCs could engraft injured kidneys. We administered DsRed UDCs by tail vein injection to animals after AKI or to those that underwent sham surgery alone. Using FACS, we identified significantly more uterine cells in kidneys after ischemia–reperfusion injury compared to sham surgery without kidney injury (Fig. 2a). The results were further confirmed by qRT-PCR, amplifying rDsRed mRNA (Fig. 2b); DsRed expression was low or undetectable in the kidneys of sham surgery mice and consistently higher in those with bilateral AKI. These data demonstrate the transport of UDCs through the circulation to the injured organ and successful engraftment. We also treated male mice with UDCs. The distribution of UDCs varied according to the gender, with significantly higher numbers of cells engrafted in males with bilateral kidney injury than females with bilateral kidney injury (Fig. 2c).

We next compared bilateral to unilateral kidney injury and demonstrated that the UDCs preferentially engraft the injured kidney. When UDCs were injected in mice with unilateral renal ischemia–reperfusion injury, the numbers of DsRed positive cells were significantly higher in the injured kidneys than in those without the injury (Fig. 2d**)**. The UDCs specifically targeted the injured kidney.

The findings were further evaluated using in vivo imaging and fluorescence-activated cell sorting. In vivo imaging in mice with unilateral renal ischemia–reperfusion injury and UDCs injection clearly indicate the presence of the DsRed positive cell population only in injured kidneys (Fig. 2e). Similarly, fluorescence-activated cell sorting shows the absence of DsRed cells in the non-injured kidneys (Fig. 2f) and indicates the presence of a DsRed positive cell population in injured kidneys (Fig. 2g).

### UDCs contribute to the repair of the injured kidney

**Fig. 3:**
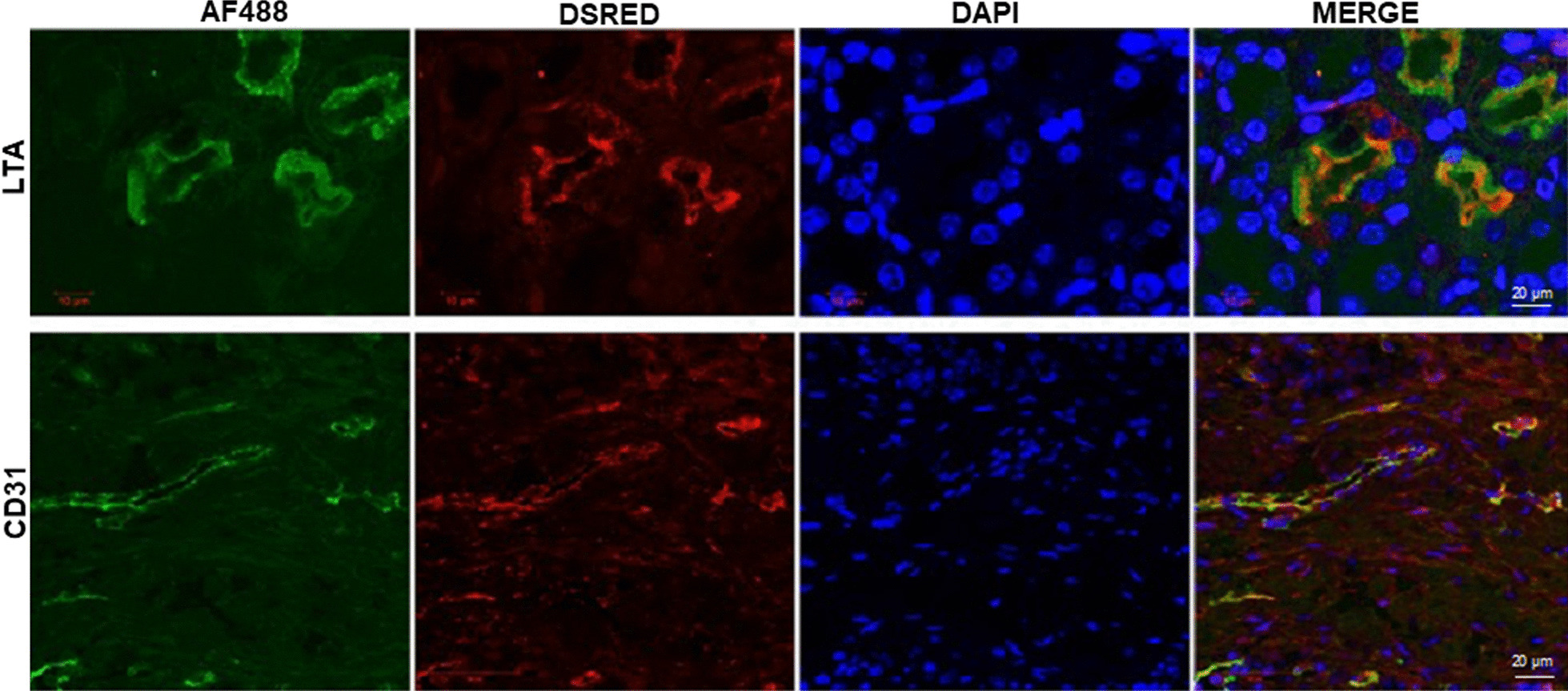
Immunofluorescence staining of LTA, CD31, DAPI and DsRed in ischemia–reperfusion injured kidney. Colocalization of DsRed with either LTA or CD31 shows incorporation of endometrial stem cells into the renal tubules or vasculature, respectively. Scale bar: 50 μm

Repair of ischemic injury requires regeneration of damaged renal tubules as well as vascular repair. The kidneys from mice with the renal ischemia–reperfusion injury and tail vein injection of DsRed positive UDCs were extracted and stained with Lotus tetragonolobus agglutinin (LTA) and CD31 to evaluate the contributions of UDCs in renal tubule regeneration and blood vessel formation, respectively. Immunofluorescence staining detected LTA positive tubular cells in injured kidneys. Triple staining with LTA, DsRed, and DAPI resulted in triple positive staining cells. Triple staining with CD31, DsRed, and DAPI also yielded positive results (Fig. 3). Together these results show a role for UDCs in tubule regeneration and revascularization.

### Kidney function and survival analysis

**Fig. 4:**
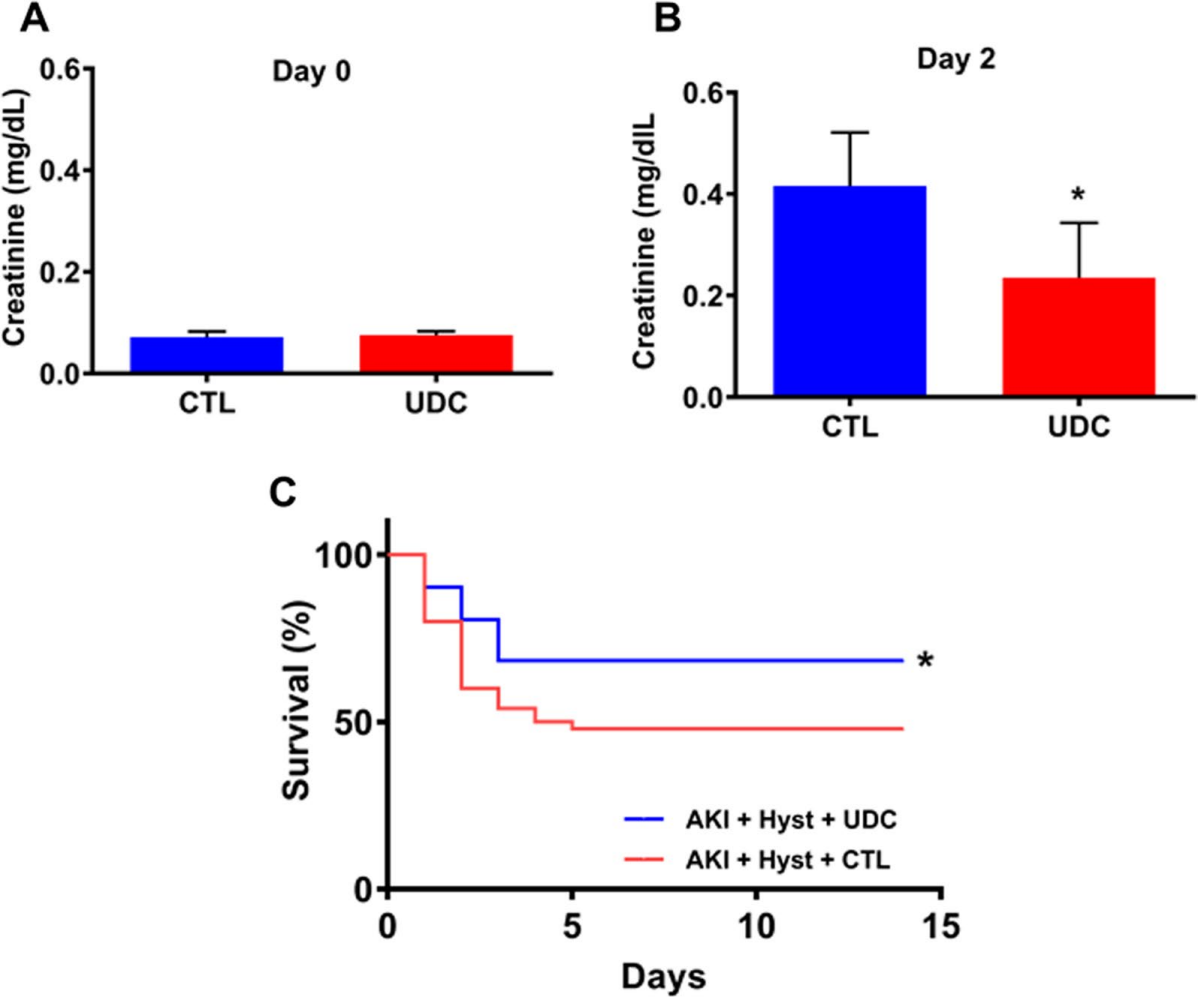
Serum creatinine levels and survival analysis. **a** Day 0 and **b** Day 2 serum creatinine levels in the DsRed positive uterine-derived cells (UDCs) treated group compared to controls, after renal ischemia–reperfusion injury. Each bar represents the mean ± SEM of two individual experiments and each experiment was performed in triplicate). *denotes statistical significance (*p* < 0.05) control vs UDC. **c** Survival analysis of control vs UDC treatment groups treatment groups; AKI indicates kidney with ischemia–reperfusion injury; Survival was improved after UDC treatment. *denotes statistical significance (*p* < 0.05) AKI + Hyst + CTL vs AKI + Hyst + UDC

To evaluate the effects of UDCs on kidney function after AKI, blood samples were collected and serum creatinine levels measured. On day 0, the day of bilateral renal ischemia–reperfusion injury, there were no significant difference in serum creatinine levels between the control group and the UDC injection group as shown in Fig. 4a. However, compared to the control group, serum creatinine levels of mice with UDCs injection showed significantly lower serum creatinine level on Day 2 (0.22 mg/dl vs 0.45 mg/dl, twofold; *p* = 0.02) as shown in Fig. 4b.

We further analyzed survival of hysterectomized mice with bilateral renal ischemia–reperfusion injury between the control and UDCs injection groups. The survival rate was significantly higher in the group that received UDCs compared to controls after 2 weeks (68% vs 48%, respectively; *p* = 0.04) (Fig. 4c).

In addition to day 2, we measured the creatinine levels at multiple time points until 3 weeks post-injury; however, continued measurements were limited to those mice that survived. In all survivors, the creatinine levels improved over time and the differences between groups narrowed as more mice with higher creatine levels died in the untreated group.

### Effects of UDCs on cytokine expression in the injured kidney

**Fig. 5:**
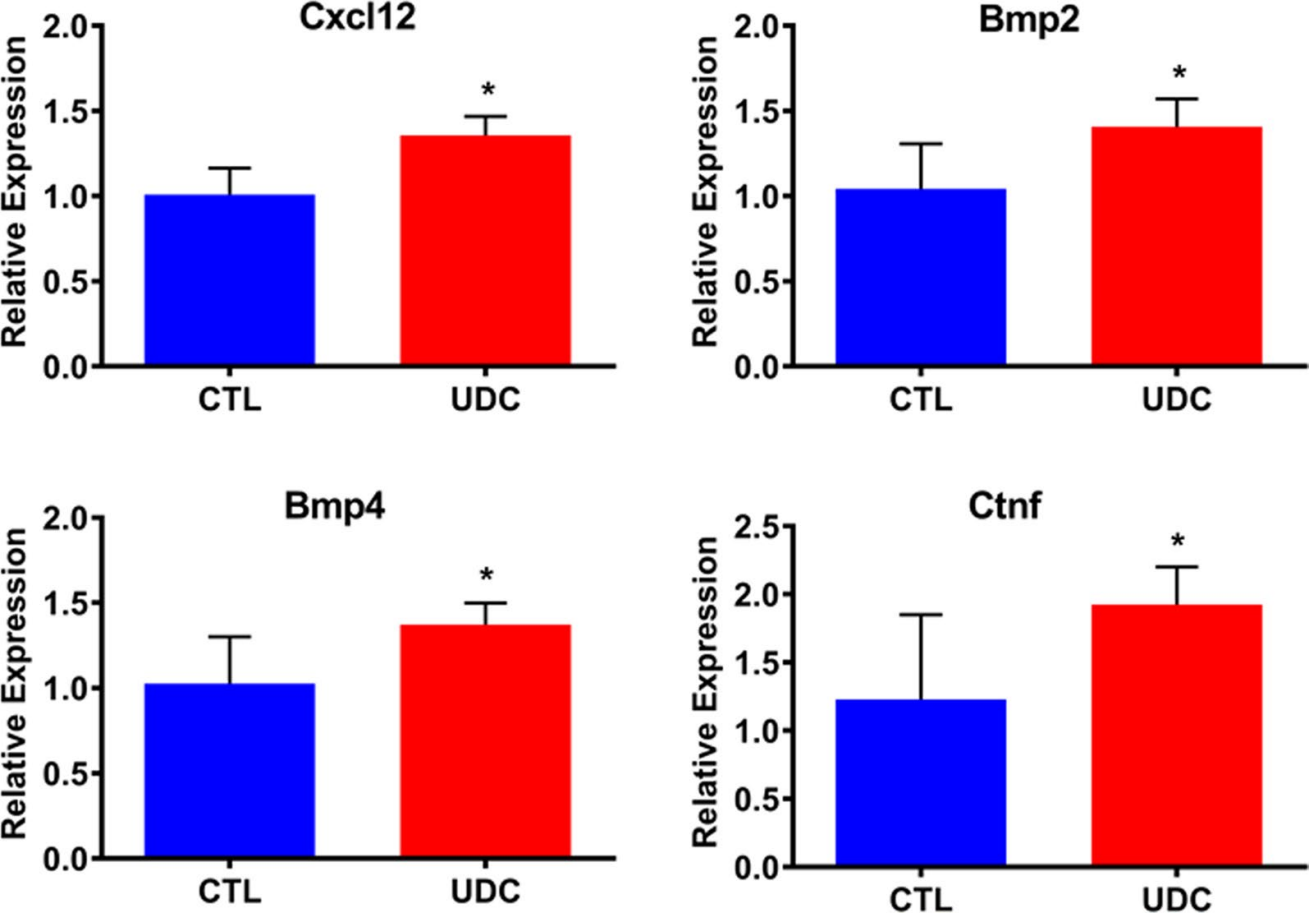
Renal mRNA expression by qRT-PCR. *Cxcl12*, *Bmp2*, *Bmp4* and *Ctnf* in the renal ischemia–reperfusion injury after treatment with DsRed positive uterine-derived cells (UDCs) vs control. *N* = 8 mice per group. Each bar represents the mean ± SEM of three individual experiments and each experiment was performed in duplicate). *denotes statistical significance (*p* < 0.05) control vs UDC

Cytokines that are related to kidney regeneration and stem cell activity were evaluated in the injured kidneys after UDCs or control injections as shown in Fig. 5. Expression of *Cxcl12*, a known mesenchymal stem cell chemotactic, was significantly higher in injured kidneys after UDCs injection than controls. Expressions of *Bmp2* and *Bmp4*, known to be associated with kidney development and repair [18–20], were also shown to be elevated in UDCs treated group compared to controls. Ctnf, a growth factor known to be involved in renal tubular repair processes after ischemia–reperfusion injury [21], displayed significantly higher expression in renal tissue of the UDC injection group than in controls.

## Discussion

In this study, we evaluated the potential roles of UDCs in regeneration of renal tubules and kidney function after AKI. We successfully established an AKI mouse model by inducing renal ischemia–reperfusion injury. UDCs were shown to travel through the circulation in order to target and successfully engraft to the injured kidney. Furthermore, UDCs are involved in renal tubule regeneration and angiogenesis by directly contributing to mesenchymal stem cell differentiation, as well as increasing angiogenic factors and BMPs. Most importantly, UDCs treatment had beneficial effects on renal function and led to increased survival of mice after AKI.

Although AKI is associated with increased morbidity and mortality, renal function can be recovered; recent studies emphasize the importance of the renal tubular cells during the recovery process [22–27]. The renal tubule has an extraordinary capacity to undergo regeneration within a few days after AKI, and the sources of these regenerating cells still remain unclear and controversial. Berger et al. showed that recovery from AKI involved intrinsic tubular cells and these intrinsic tubular cells can arise from any surviving tubular cells rather than from a fixed progenitor cell population [28]. Others have noted that extrinsic cells from the bone marrow or mesenchymal stem cells are not required for tubular recovery [29, 30]. However, there are numerous studies indicating the benefits of therapy with mesenchymal stem cells in the recovery process of AKI through various mechanisms, including anti-inflammation, anti-apoptosis, antifibrosis, immunomodulation, and proangiogenesis [31]. These mesenchymal stem cells are derived from many different tissues and organs, such as dental pulp [32], amniotic fluid [33], umbilical cord [34], and bone marrow [35, 36]. Our studies on uterine stem cells are further supported by a recent study showing that uterine-derived feline mesenchymal stem cells (UMSCs) have a therapeutic effect on chronic kidney disease (CKD) in cats [37]. Uterine-derived stem cells may be beneficial in the acute or chronic disease state. Further, exosomes derived from induced multipotent stems have therapeutic effect on AKI, suggesting that the identification of molecular mediators may reduce the need for cell-based therapy [38]; further studies to determine the relative efficacy are indicated. All point to a therapeutic use of stem cells in the treatment of renal disease.

Although promising, use of mesenchymal stem cells from some tissues and/or organs has several limitations. To prevent the risk of immune rejection, autologous mesenchymal stem cell transplantation may be ideal but is a complex and time-consuming process with relatively high costs. Obtaining stem cells from most sources requires general anesthesia and an operative procedure. In this regard, UDCs, specifically endometrial stem cells, have many advantages over other sources. They are easily accessible in the outpatient setting with minimal or no analgesia required (endometrial biopsy). The endometrium is additionally a self-renewing tissue. Endometrial stem cells can also be expended in vitro with vast differentiation potential in vivo and in vitro [39].

In this study, we have shown that UDCs contribute to regeneration of renal tubules and improve kidney function in mice with renal ischemia–reperfusion injuries; our data support a potential role for mesenchymal stem cells in repair of damaged kidneys. Our findings are in line with previous work showing human endometrial regenerative cells from menstrual blood attenuated renal ischemia–reperfusion injury in mice by anti-inflammatory and immune-regulatory effects [40]. Interestingly, our data strongly suggest UDCs as one of the origins of tubular regeneration and recovery, which was confirmed by double positive staining of LTA and DsRed. The data also suggest that UDCs contain various cytokines related to tubule repair, stem cell homing, and angiogenesis, which promoted new vessel formation in the injured kidneys.

We further evaluated the renal function and survival rate of the mice with injured kidneys after UDCs injection and noted improvement in serum creatinine levels 48 h after treatment, with increased survival after 2 weeks. These findings are supported by a recent systematic review involving 21 studies indicating improved outcomes in impaired renal function with the use of mesenchymal stem/stromal cells therapy in small animal models [41].

Since it has been suggested that renal ischemia–reperfusion injury involves cellular damage caused by ischemia as well as delayed renal damage resulting from inflammatory and immune responses [42], numerous studies have focused on inflammation and immune activations in renal ischemia–reperfusion injury models. These studies have shown that activation and infiltration of neutrophils, macrophages, and other inflammatory mediators are involved in modulating severity of injury in experimental models [43–45]. In this study, particular emphasis was made on the role of mesenchymal stem cells and their recruitment after renal ischemia–reperfusion injury. The results showed that injection of UDCs increased expression of *Cxcl12* and BMPs along with *Ctnf*. The increase in Ctnf levels is known to parallel the recovery of renal structure following ischemia–reperfusion injury [21]. Various cytokines related to mesenchymal stem cell recruitment and differentiation also play a significant role in the healing process after renal ischemia–reperfusion injury.

Interestingly, the survival analysis revealed differences after renal ischemia–reperfusion injury according to the presence or absence of the uterus. Hysterectomized mice had a much lower survival rate than those with intact uteri after 2 weeks, suggesting the importance of the uterus and resident endometrial stem cells for renal regeneration after AKI. Similarly, we saw more exogenous uterine-derived cells engrafting the male kidneys, likely due to the absence of a competing endogenous source of these cells from a uterus. These results are consistent with the higher incidence of acute renal failure in males compared to females based on the analysis of hospitalized Medicare beneficiaries and a prospective multicenter community-based study [46, 47]. There are approximately 600,000 hysterectomies performed yearly in the USA alone. Diminished renal function after hysterectomy has been historically attributed to unrecognized injury or obstruction of the ureter. Our results suggest another possible explanation based on the loss of resident stem cells that may contribute to renal repair after injury. Hysterectomy without oophorectomy has traditionally been considered a low-risk surgery with no long-term consequences. However, several recent studies have linked hysterectomy to an increased incidence of renal cell carcinoma [48–50]. Endometrial stem cells may also have a role in preserving renal tumor surveillance. Further studies are needed to explore the non-gynecologic consequences of hysterectomy, especially the effect on kidneys and other embryologically related tissues. Hysterectomy should no longer be considered a procedure void of long-term medical consequences.

## Conclusions

We have shown that UDCs engraft injured kidneys following ischemia–reperfusion injury and contribute to proximal tubule and vascular regeneration, with resultant improvement in kidney function and survival. Evidence of a uterine contribution to tissue repair emphasizes the importance of the uterus as a source of stem cells and tissue regeneration. This finding has direct clinical implications considering the high rate of hysterectomy in the USA. While further validation is needed, the utilization of UDCs is also a potential therapeutic option for AKI treatment.

## Data Availability

Data available with Ramanaiah Mamillapalli and Hugh S Taylor.
